# Glycyrrhizin Represses Total Parenteral Nutrition-Associated Acute Liver Injury in Rats by Suppressing Endoplasmic Reticulum Stress

**DOI:** 10.3390/ijms140612563

**Published:** 2013-06-14

**Authors:** Jai-Jen Tsai, Hsing-Chun Kuo, Kam-Fai Lee, Tung-Hu Tsai

**Affiliations:** 1 Division of Gastroenterology, Department of Medicine, National Yang-Ming University Hospital, I-Lan 26042, Taiwan; E-Mail: jjthsai@yahoo.com.tw; 2 Institute of Traditional Medicine, School of Medicine, National Yang-Ming University, Taipei 11221, Taiwan; 3 Department of Nursing, Chronic Diseases and Health Promotion Research Center, Chang Gung University of Science and Technology Chiayi Campus, Chiayi 61363, Taiwan; E-Mail: guscsi@gmail.com; 4 Department of Pathology, Chang Gung Memorial Hospital at Chiayi, Chiayi 61363, Taiwan; E-Mail: lkf2002@adm.cgmh.org.tw; 5 Graduate Institute of Acupuncture Science, China Medical University, Taichung 40402, Taiwan; 6 Department of Education and Research, Taipei City Hospital, Taipei 10341, Taiwan

**Keywords:** total parenteral nutrition (TPN), endoplasmic reticulum (ER), glycyrrhizin

## Abstract

Total parenteral nutrition (TPN) is an artificial way to support daily nutritional requirements by bypassing the digestive system, but long-term TPN administration may cause severe liver dysfunction. Glycyrrhizin is an active component of licorice root that has been widely used to treat chronic hepatitis. The aim of this study is to investigate the hepatoprotective effect of glycyrrhizin on TPN-associated acute liver injury *in vivo*. Liver dysfunction was induced by intravenous infusion of TPN at a flow rate of 20 mL/kg/h for three h in Sprague Dawley rats. The rats were pretreated with Glycyrrhizin (1, 3 and 10 mg/kg intravenously). After receiving TPN or saline (control group) for three h, the rats were sacrificed, blood samples were collected for biochemical analyses and liver tissue was removed for histopathological and immunohistochemical examination. We found that aspartate aminotransferase (AST), alanine aminotransferase (ALT), total bilirubin (TB) and triglyceride (TG) levels were significantly increased in the TPN group without glycyrrhizin pretreatment and decreased in the glycyrrhizin-pretreated TPN group in a dose-dependent manner. The stained liver sections showed that glycyrrhizin relieved acute liver injury. The upregulation of serum protein biomarkers of reactive nitrogen species, including nitrotyrosine and inducible NO synthase (iNOS), were attenuated by glycyrrhizin pretreatment. Levels of endoplasmic reticulum (ER) stress factors, such as phosphorylation of JNK1/2, p38 MAPK and CHOP, were decreased by glycyrrhizin pretreatment. In summary, our results suggest that glycyrrhizin decreases TPN-associated acute liver injury factors by suppressing endoplasmic reticulum stress and reactive nitrogen stress.

## 1. Introduction

Total parenteral nutrition (TPN) is a method for providing nutrients, such as glucose, amino acids, lipids, added vitamins and dietary minerals, to patients who are unable to sustain adequate nutrition by standard enteral means, namely, those who suffer from gastrointestinal disorders. However, long-term TPN administration increases the risks of hyperglycemia, hepatic inflammation, steatosis, insulin resistance and reduced immune responses [1,2]; among these, hepatitis is one of the most frequent complications [3,4]. Although several factors have been associated with a high risk of chronic TPN-induced hepatitis [5], such as enteral feeding history, septic events, length of intestinal resection and prematurity/low birth weight, its pathogenic mechanism is not fully understood [6,7].

Previous studies demonstrated three possible routes of pathogenesis for chronic TPN-induced hepatitis: direct toxic effect of detergent-like bile salts [8–14], increasing oxidative stress [15–20] and hepatocyte apoptosis [21–23]. According to Loff *et al.* and Shamir *et al.*, injury to liver acinar Zone 3, which is composed of hepatocytes located around the hepatic venule that are mainly involved in the production of bile salt, was found in TPN related hepatitis in animal models [12,13]. TPN-related hepatic injury in Zone 3 might be a typical feature for the direct toxic effect of the bile salt [13,14]. Moreover, TPN is an important source of oxidants and peroxides that are derived mainly from the reduction of dissolved oxygen by electron donors, such as vitamin C, amino acids and lipids [16]. Peroxide-induced oxidative stress from TPN can trigger pro-inflammatory cytokines and activate inflammatory cytokine cascades [15,16]. Cytokines can be negatively regulated by Suppressors of Cytokines Signaling (SOCS), which is a protein that is highly upregulated in response to pro-inflammatory cytokines [18,19]. In addition, hepatocyte apoptosis has been observed at 24 h after TPN administration in a mouse model and is correlated with the increased expression of Fas, the pro-apoptotic factor Bad and the anti-apoptotic factor Bcl-xl [21]. Katz’s group also observed that administering TPN to mouse for 14 days increased hepatocyte apoptosis in bowel resection mouse [24]. Hepatocytes are very susceptible to endoplasmic reticulum (ER) stress, and ER-stressed hepatocytes are more susceptible to tumor necrosis factor-α-induced cell death and apoptosis [25].

**Figure 1:**
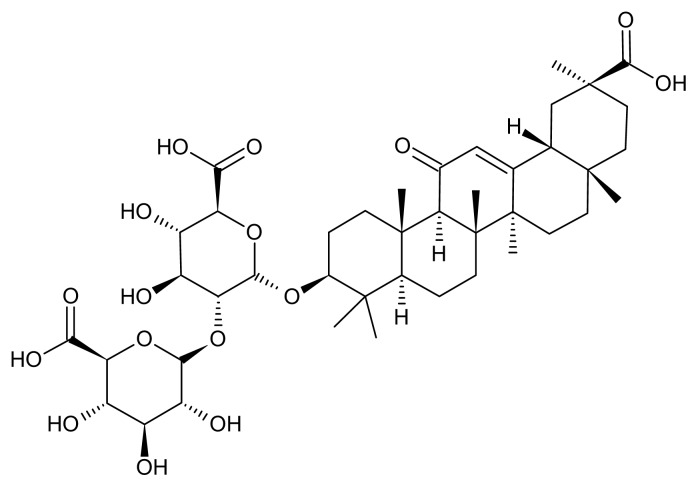
The chemical structure of glycyrrhizin (GL).

Recently, glycyrrhizin has been reported to exhibit hepatoprotective effects in the treatment of viral hepatitis [26]. Glycyrrhizin (GL; Figure 1) is an active compound isolated from the root of licorice (Glycyrrhiza glabra; Glycyrrhiza radix; Chinese name: Gancao). In traditional Chinese medicine, licorice is one of the most commonly used herbs in treating liver disorders [27]. It is also used to reduce toxicity, to improve appetite and enhance the effectiveness of other ingredients in prescriptions [27,28]. There is evidence for the therapeutic application of glycyrrhizin for chronic hepatitis induced by viral infections, toxin exposure and ischemic-reperfusion injury [29]. In Japan, Stronger Neo-Minophagen C (SNMC), a commercially available glycyrrhizin-containing prescription, has been adopted in clinical practice to treat chronic viral hepatitis for over 30 years [30,31].

In this study, we used a TPN-induced acute liver injury rat model to evaluate the hepatoprotective effect of glycyrrhizin. We assessed the early stage of TPN-related acute liver injury by biochemical, histopathological and immunohistochemical investigation to understand the possible molecular mechanism. We found that glycyrrhizin exhibits hepatoprotective activity in TPN-induced acute liver injury by suppressing endoplasmic reticulum (ER) stress and pro-inflammatory cytokines.

## 2. Results

### 2.1. Biochemical Analysis of Glycyrrhizin

To investigate the effect of TPN on biochemical markers of acute liver injury in experimental rats, TPN was intravenously infused via the left femoral vein of rats at a flow rate of 20 mL/kg/h for three hours. After GL treatment, serum ALT and AST levels were significantly elevated compared to the control group (rats with normal saline infusion and without GL pretreatment, Table 1). Importantly, these enzyme levels were reduced in TPN rats pretreated with glycyrrhizin in a dose-dependent manner (1, 3 and 10 mg/kg, i.v.). The most significant effect was observed in the group of TPN rats that received glycyrrhizin (10 mg/kg) pretreatment, as the ALT and AST concentrations in the serum of these rats were restored to the levels of the saline-control group.

Total bilirubin (TB) also decreased in a dose-dependent manner in response to glycyrrhizin pretreatment (1, 3 and 10 mg/kg, i.v.), but the lowest dose of glycyrrhizin (1 mg/kg, i.v.) had no effect (Table 1). Similar dose-response patterns of glycyrrhizin pretreatment were observed on triglyceride (TG) levels (Table 1).

### 2.2. Effect of Glycyrrhizin on Hepatic Histopathological Changes

**Figure 2:**
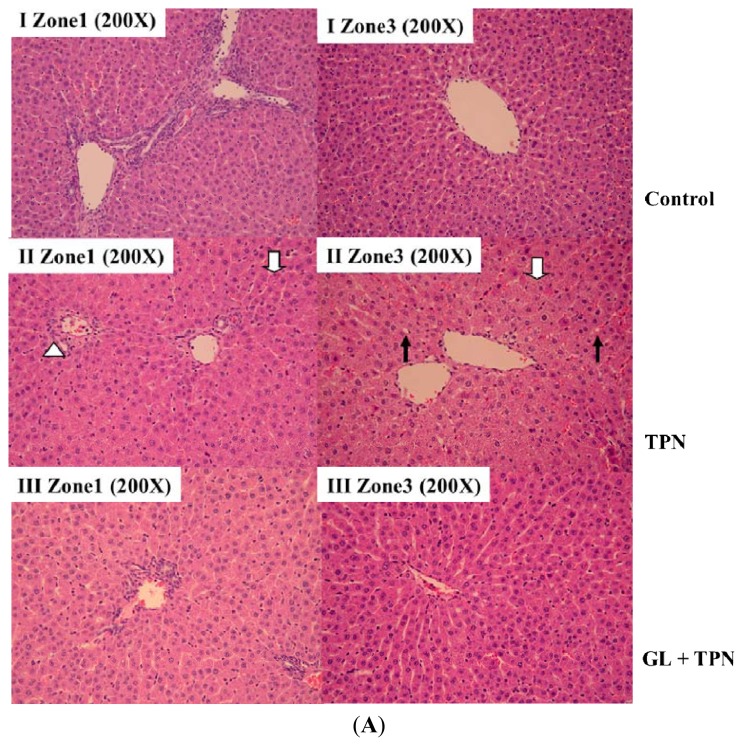

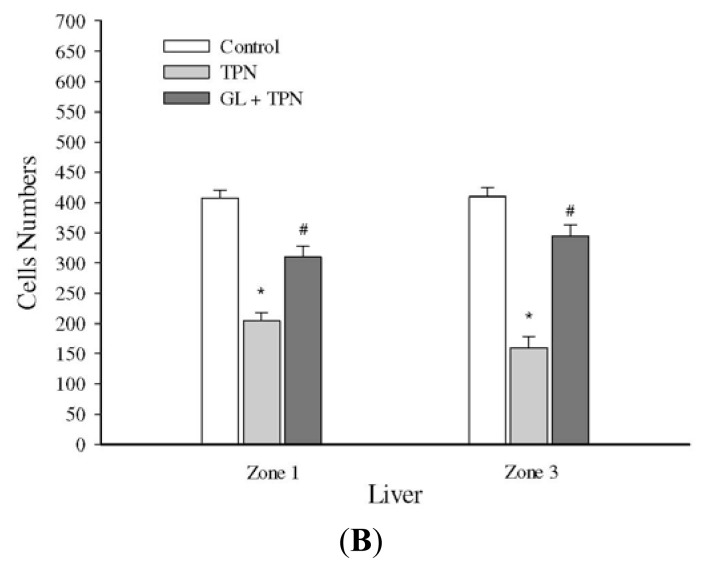
Histological examination of the effect of glycyrrhizin (GL) on total parenteral nutrition (TPN) rats. The livers were H&E-stained to examine the portal (zone) and terminal hepatic venules (Zone 3). (**A**) Saline infusion control (I); TPN infusion without treatment (II); and GL pretreatment (10 mg/kg) before TPN infusion (III) staining of Zone 1 (left column) and Zone 3 (right column) of the liver. Liver pathology in the control group exhibited normal hepatocyte structure and arrangement. The TPN non-treated group exhibited damaged liver structure, as indicated by monocyte infiltration (open arrowhead), steatosis (filled arrow) and the presence of necrotic cells (open arrow). Pretreatment with GL (10 mg/kg) inhibited hepatocyte damage; (**B**) Quantitative evaluation of pathological examinations. Normal hepatocytes were counted from 10 random fields (200× magnification) of each liver sample and the values represent the mean ± SD of six rats. *p < 0.05, compared with control group, ^#^*p <* 0.05 between pretreatment groups and TPN group.

To investigate the histopathological changes in response to GL, liver tissues were fixed in 10% natural buffered formalin and then stained with H&E. When comparing the liver cell morphology of the control, TPN and TPN pretreated with glycyrrhizin (10 mg/kg) groups, only the control and glycyrrhizin-pretreated group exhibited normal liver cell structure, with a well-defined cytoplasm and nucleus in the cells and ribbon-like hepatocyte arrangements (Figure 2A). In contrast, the liver cells in the TPN rats, particularly in Zone 3 of the portal venule tract, appeared as feather-like hepatocyte arrangements (open arrow Figure 2A). In addition, the hepatic parenchymal cells (hepatocytes) of the TPN rats exhibited signs of hepatic steatosis, such as an excess accumulation of fat (triglycerides) (filled arrow in Figure 2A).

The quantification of pathological liver cells revealed that the number of normal hepatocytes was reduced in the TPN group [TPN *vs.* control in Zone 1 (*T* = 204 ± 14; control = 408 ± 12, *p <* 0.05) and Zone 3 (*T* = 160 ± 19; control = 410 ± 14, *p <* 0.05), see Figure 2B], and there was an increase in the cell in the group pretreated with glycyrrhizin (10 mg/kg) compared with the TPN group. [GL + TPN *vs.* TPN in Zone 1 (*T* = 204 ± 14; GL = 310 ± 18, *p <* 0.05) and Zone 3 (*T* = 160 ± 19; GL = 344 ± 19, *p <* 0.05, see Figure 2B)].

### 2.3. Effects of Glycyrrhizin on the Expression of Nitrotyrosine, iNOS and Proinflammatory Cytokines (IL-1β, IL-6 and TNF-α)

**Figure 3:**
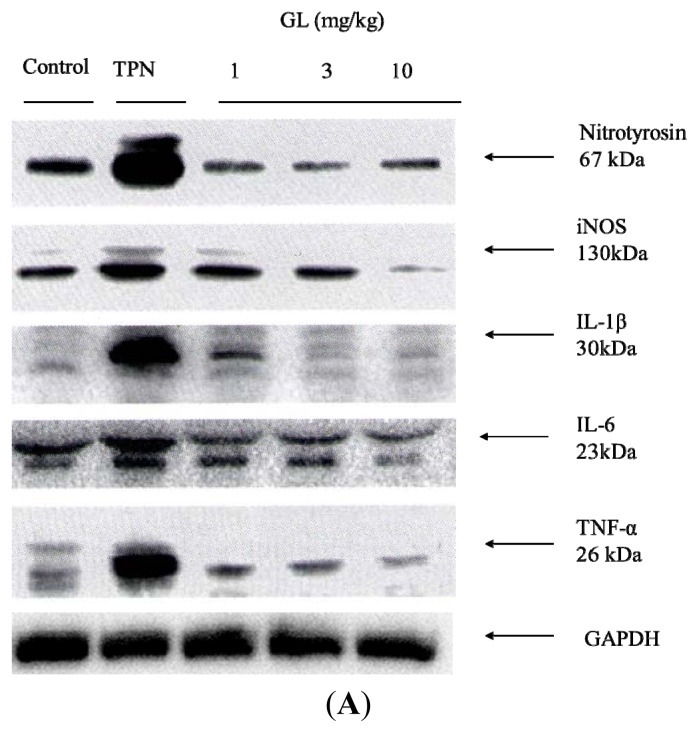

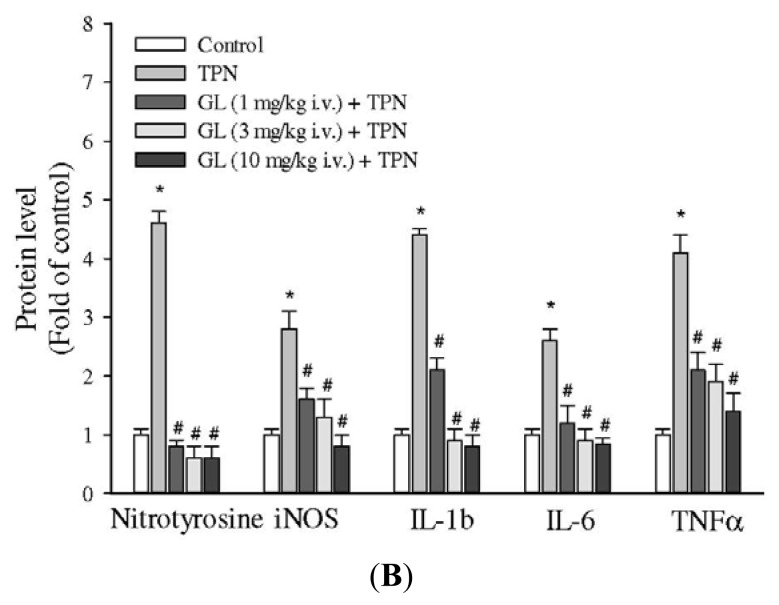
The relative intensity of RNS oxidants and acute inflammation cytokines in rat serum. (**A**) Representative Western blot analysis of the following inflammatory cytokine proteins in rat serum: nitrotyrosine, iNOS, IL-1β, IL-6 and TNF-α. The TPN group had high levels of oxidative stress and inflammation; (**B**) The relative density was calculated by dividing the protein density by the density of all the matched spots in the respective gel. The expression of RNS oxidants and inflammatory proteins in the serum were higher in the TPN group compared to the control group and pretreatment groups. GAPDH served as an internal control. The protein levels were quantified using densitometric analysis, and the control was set at 100%. The data depicted in bar graph represent the mean ± SD of three independent measurements. (*n =* 6 in each group). *p < 0.05, compared with control group, ^#^*p <* 0.05 between the pretreatment groups and the TPN group.

After TPN administration, serum protein biomarkers for reactive nitrogen species, nitrotyrosine and inducible NO synthase (iNOS) were increased in the TPN group compared with both the control and three GL-pretreated groups (Figure 3A). Moreover, pretreatment with glycyrrhizin reduced the expression of TNF-α, iNOS and nitrotyrosine proteins in a dose-dependent manner (Figure 3B). In particular, the highest dose (10 mg/kg) resulted in the most powerful inhibition of iNOS and nitrotyrosine (Figure 3B). These results demonstrated that TPN administration increased the production of proinflammatory cytokines, including IL-1β, IL-6 and TNF-α, and these increases were inhibited by pre-treatment with glycyrrhizin.

### 2.4. Anti-Apoptotic Effects of Glycyrrhizin

**Figure 4:**
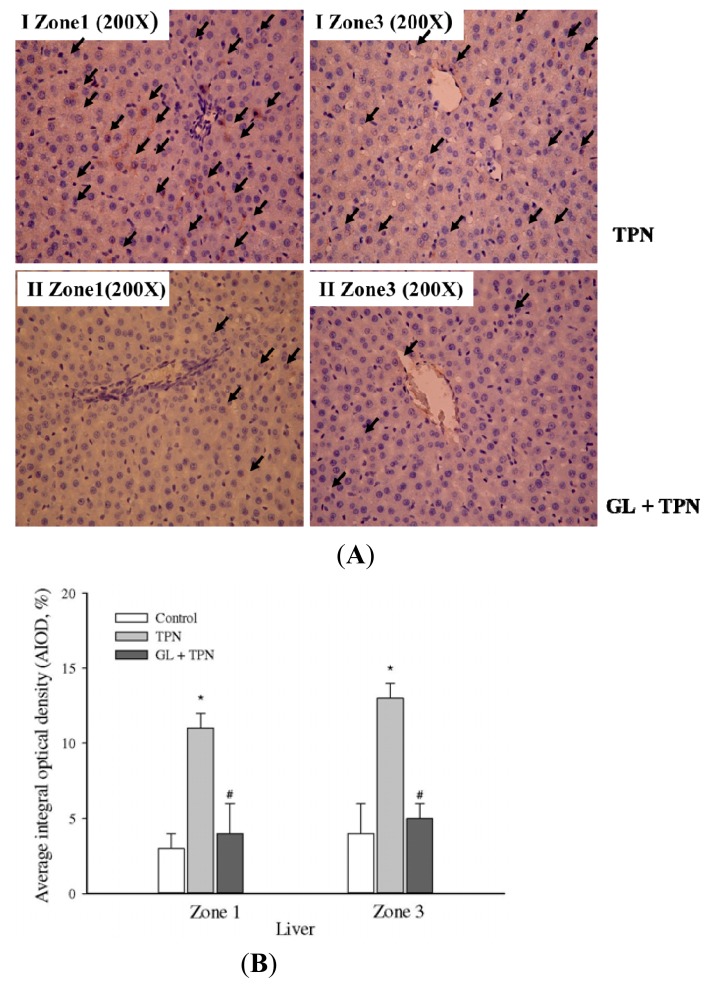
Immunohistochemical analysis of cleaved caspase-3 staining in rat livers from different liver zonal areas. TPN *versus* GL pretreatment group (**A**) Pretreatment GL (10 mg/kg) prior to TPN administration. The TPN group (I) and GL pretreatment group (II) animals received an equal volume of solvent. Group (I) normal saline 1 mL, i.v. bolus before TPN administration; pretreatment group (II) GL (10 mg/kg) in normal saline 1 mL, i.v. bolus before TPN administration. Representative photographs after immunostaining with antibodies against cleaved caspase-3; (**B**) Quantitative analysis of immunohistochemical staining of cleaved caspase-3. Average integrated optical density (AIOD) of the positively stained area was evaluated from three randomly selected observation fields in each liver section. The data are expressed as the mean ± SD (*n =* 6 per group). *p < 0.05, compared with the control group, ^#^*p <* 0.05 between pretreatment groups and TPN group.

To determine what kind of cell death was involved in the TPN related acute liver injury, the presence of the apoptotic marker cleaved caspase-3 was examined in liver tissues from the control, TPN and pretreatment with glycyrrhizin groups. As shown in Figure 4, the results demonstrated that pretreatment with glycyrrhizin prevented hepatocyte apoptosis in TNP-related hepatitis rat model. The results revealed a distinct staining of cleaved caspase-3 within the hepatocyte in TPN group (Figure 4A). There was a significant and nearly three-fold increase in hepatocyte apoptosis (as determined by cleaved caspase-3 staining) in Zone 1 in the TPN group (11 ± 1) compared with both the control (3 ± 1; *p <* 0.05) and GL (10 mg/kg) pretreatment groups (4 ± 2; *p <* 0.05) (Figure 4B). In Zone 3, there was also an approximately three-fold increase in hepatocyte apoptosis in the TPN group (13 ± 1) compared with both the control (4 ± 1; *p <* 0.05) and GL (10 mg/kg) pretreatment groups (5 ± 1; *p <* 0.05). These results indicated that TPN induced liver apoptosis, whereas GL pretreatment reduced liver apoptosis.

### 2.5. Effects of Glycyrrhizin on the Phosphorylation of JNK1/2 and p38, as well as CHOP Expression

**Figure 5:**
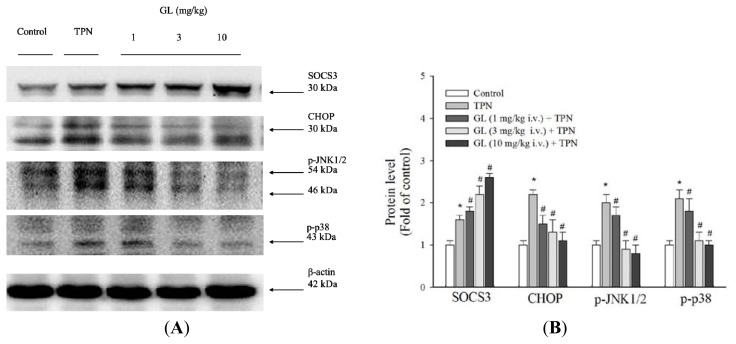
The effect of glycyrrhizin (GL) on liver histological SOCS3 and apoptotic pathway protein expression in rat livers. (**A**) Western blot analysis of proteins related to SOCS3 and the apoptotic pathway in the control, TPN and GL-pretreated groups; (**B**) SOCS3, CHOP and MAPK signaling protein expression were measured. The TPN group significantly increased SOCS3 protein expression, whereas SOC3 protein expression was decreased in the GL pretreatment groups. The phosphorylated of p38 MAPK and JNK proteins was increased in the TPN groups compared to the control and pretreatment groups. CHOP protein was also increased following TPN treatment. Equal amounts of protein from the total cell lysates of rat livers pretreated with GL were analyzed. β-actin served as an internal control. Protein levels were quantified using densitometric analysis and the control was set at 100%. The data depicted in bar graph are the mean ± SD of three independent measurements (*n =* 6 in each group). *p < 0.05, compared with control group, ^#^*p <* 0.05 between pretreatment groups and TPN group.

To investigate the molecular mechanism that underlies the pharmacological effects by which glycyrrhizin prevents TPN-induced hepatocyte apoptosis, we assessed the expression of several proapoptotic molecules under endoplasmic reticulum (ER) stress. These molecules included Apoptosis Signal-regulating Kinase 1 (ASK1), c-Jun NH2-terminal Kinase (JNK1/2), p38 MAPK and CHOP [22,23,32,33]. As shown in Figure 5A, the administration of TPN for three h remarkably increased the phosphorylation of JNK1/2 and p38 MAPK, as well as the expression of CHOP. Conversely, pretreatment with glycyrrhizin (1, 3 and 10 mg/kg) reduced the phosphorylation of JNK1/2 and p38 MAPK. In addition, glycyrrhizin inhibited CHOP protein activation in a dose-response manner (Figure 5B), and the strongest inhibition occurred after pretreatment with 10 mg/kg glycyrrhizin.

### 2.6. Inhibitory Effect of Glycyrrhizin on Inflammation via Activation of SOCS3 in TPN-Related Acute Liver Injury *in Vivo*

**Figure 6:**
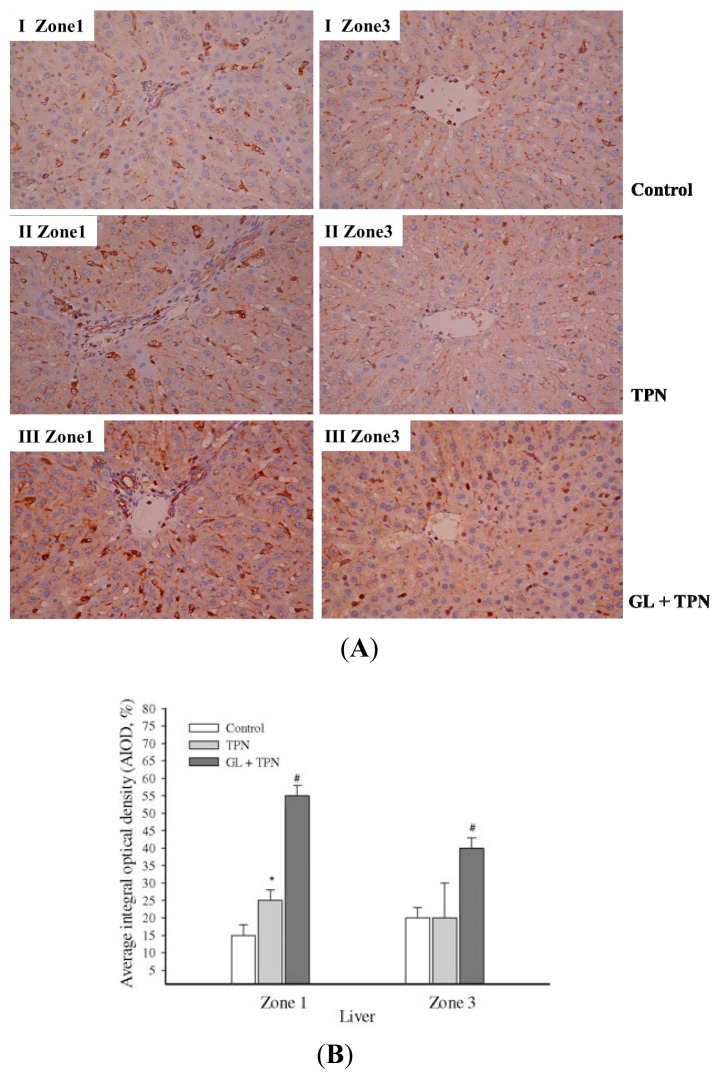
The effect of glycyrrhizin (GL) on liver cytokine-inducible SOCS3 protein levels. (**A**) Immunohistochemical staining for SOCS3 in rat livers was analyzed in different liver zonal areas: control rats (I); rats with TPN infusion (II); GL-pretreated rats (10 mg/kg) (III) staining of the portal and terminal hepatic venues zones; (**B**) The quantitation of immunohistochemical staining of SOCS3 levels by average integrated optical density (AIOD). The positive stained area was evaluated from three randomly selected observation fields of each liver section. The data represent the mean ± SD (*n =* 6/group). *p < 0.05, compared with control group, ^#^*p <* 0.05 between the pretreatment and TPN groups.

To evaluate the anti-inflammatory effect of glycyrrhizin, the protein level of SOCS3 in rat livers was examined. SOCS3 is an inducible negative feedback regulator of inflammation. As shown in Figure 5A, the protein level of SOCS3 increased after three h of TPN solution administration, and pretreatment with glycyrrhizin significantly increased the SOCS3 protein level in a dose-dependent manner (Figure 5B). Endogenous SOCS3 was induced after TPN administration to antagonize the inflammation process. Furthermore, GL pretreatment increased the expression of SOCS3 (Figure 5A), suggesting that this treatment effectively blocked the liver inflammatory cascade. We also investigate the immunochemical activation of SOCS3 in portal Zone 1 and Zone 3, which is close to the terminal hepatic venule tracts (Figure 6A). These data were validated by quantitative evaluation of SOCS3 protein intensity using the average integral optical density (Figure 6B).

## 3. Discussion

In contrast to most TPN-related hepatitis investigations, which focus on late stage liver injury, we established an animal model of TPN-related acute liver injury and provide valuable implications for essentially preventing TPN-induced hepatitis. Our data provide evidence that suppressing endoplasmic reticulum stress and reactive nitrogen stress may have hepatoprotective effects and reduce the development of TPN related hepatitis.

As shown in Table 1, ALT, AST and triglyceride levels were elevated in rat serum 3 h after TPN administration, and pretreatment with GL (1, 3 and 10 mg/kg) normalized these levels. Figure 2A shows the presence of severe hepatocyte steatosis in the TPN group. The liver is the main organ for glucose disposal in the normal animals, which received 45% TPN-derived glucose [34]. The overconsumption of glucose is associated with the increased accumulation of triglycerides in hepatocytes. As shown in Figure 2A, glycyrrhizin improved the extent of accumulation. However, the difference in total bilirubin between the GL pretreatment group (1 mg/kg) and the TPN group was not significantly different, suggesting that an adequate therapeutic dose was achieved, with only the ALT and AST concentrations being affected, but not total bilirubin levels (Table 1). Based on the observed biochemical and pathological data, we successfully established a model of acute liver injury using TPN in rats, finding that liver injury could be prevented by pretreatment with glycyrrhizin in a dose-dependent manner. Similarly, Shamir (1993) and Zahavi (2000) [13,14] showed a significant reduction in bile flow and bile salt secretion in rats after a two hour infusion of TPN at a rate of 10 mL/kg/h. The above reports allowed for us to speculate that short-term TPN infusion (2 h) at an infusion rate of 10 mL/kg/h can lead to bile stasis. Moreover, these previous studies also partially explain why the short-term (3 h at a rate of 20 mL/kg/h) TPN infusion used in the present study elevated liver function.

According to a previous study, the hepatoprotective effect of glycyrrhizin in viral hepatitis patients is due to its cytoprotective effect via the suppression of TNF-α-induced cytotoxicity and inhibition of immune-mediated cytotoxicity against hepatocytes [35–37]. In addition, glycyrrhizin has anti-inflammatory effects and attenuates the inflammatory response by inhibiting nuclear factor κB (NF-κB) and PI3K [38]. Our data in Figure 3 and Table 1 show that TPN administration induced systemic pro-inflammatory cytokine production and hepatitis. Glycyrrhizin can decrease the production of these cytokines and improve TPN-related acute liver injury.

The overproduction of nitric oxide and its derivatives have been implicated as a cause of tissue damage in response to inflammation. Pro-inflammatory mediators, such as NO, which is generated by iNOS and nitrotyrosine, are considered to be indicators of the reaction during the inflammatory response [39,40]. iNOS expression and the nitrosylation of tyrosine are induced in many different cell types in response to endotoxins and cytokines, such as gamma interferon, interleukin 1 and tumor necrosis factor alpha [41–43]. Thus, iNOS and nitrotyrosine serve as indicators of reactive nitrogen stress generated by NO [43]. In the present study, (Figure 3), we demonstrated that treatment with glycyrrhizin effectively inhibited NO production by suppressing the expression of iNOS and nitrotyrosine. These data suggest that the therapeutic effect by which glycyrrhizin treats TPN-related acute liver injury is partially due to the inhibition of NO production via the inhibition of iNOS, TNF-α, iNOS and nitrotyrosine protein levels, and pretreatment with glycyrrhizin (10 mg/kg) resulted in the most powerful inhibition of iNOS and nitrotyrosine.

Cytokines mediate various biological processes, including inflammation, apoptosis, necrosis and fibrosis [44,45]. Increased pro-inflammatory cytokines has been demonstrated in animal model of TPN [46,47]. These findings are consistent with our data, as GL decreased the production of IL-1β, IL-6 and TNF-α in a dose-dependent manner (Figure 3). The SOCS3 protein is highly upregulated in response to pro-inflammatory cytokines (*i.e*., IFN-γ, interleukin IL-1β and IL-6) [18]. SOCS3 is recognized to be the main modulator for the negative regulation of the cytokine-JAK-STAT pathway and, thus, can induce anti-inflammatory processes [20]. Previous investigations also demonstrated the SOCS3 is related to the severity of liver inflammation, and it plays a protective role in the cytokine-mediated inflammatory process by inhibiting IL-6-medicated STAT3 activation [48,49]. Figure 6 shows that endogenous SOCS3 increased after TPN administration as a negative regulator of TPN-related acute liver injury, and pretreatment with glycyrrhizin (Figure 5A,B) increased SOCS3 protein expression to enhance the negative regulation TPN-related acute liver injury. These findings indicate that the inhibitory effects of glycyrrhizin on hepatitis may be due to alterations in SOCS3 expression. In summary, our study demonstrated that glycyrrhizin-inducted expression of SOCS3 contributes to the inhibition of inflammation and reactive nitrogen stress (RNS) production.

Infusions containing lipids and glucose activate stress kinases (JNK1/2 and p38 MAPK) and increase ER stress in rats [50]. ER stress is known to induce apoptosis by the induction of CHOP, which is a protein important for ER stress-related hepatitis [51,52]. Figure 4A,B shows that caspase-3 cleavage is higher after TPN administration, and it is well established that the caspases are the executioners of apoptosis [53]. These results demonstrate that glycyrrhizin pretreatment significantly inhibits the phosphorylation of JNK and p38 MAPK, as well as CHOP protein activation, thereby suppressing hepatocyte apoptosis in TPN rats.

## 4. Experimental Section

### 4.1. Chemicals and Reagents

Glycyrrhizin (Figure 1), Reactive Nitrogen Species (RNS) scavengers (including nitrotyrosine and anti-iNOS), CHOP, a Janus kinase (JNK) inhibitor (SP600125) and a p38 inhibitor (SB203580) were purchased from Sigma (St. Louis, MO, USA). Total parenteral nutrition solution Kabiven™ was obtained from Fresenius Kabi AB (Uppsala, Sweden). Mouse monoclonal antibodies against GAPDH, β-actin, pro-inflammatory anti-TNF-α, IL-1β, IL-6 and nitrotyrosine were purchased from Santa Cruz Biotechnology (Santa Cruz, CA, USA). Rabbit antibodies against human SOCS3, cleaved-caspase-3, phospho-p38 MAPK (Thr180/Tyr182), phospho-JNK1/2 (Thr183/Tyr185), cdk1Tyr15 and a mouse monoclonal antibody against cdk1 were purchased from Cell Signaling Technology (Beverly, MA, USA). The TdT-mediated dUTP Nick End Labeling (TUNEL) kits were purchased from Roche (Mannheim, Germany). SDS, NP-40, sodium deoxycholate and protease inhibitor cocktails were purchased from Sigma (St. Louis, MO, USA).

### 4.2. Animal Experiments and Drug Treatment

Adult male Sprague-Dawley rats weighing 200 ± 20 g were obtained from the National Yang-Ming University Animal Center, Taipei, Taiwan. The rats were pathogen-free and had free access to food (Laboratory Rodent Diet 5001, PMI Nutrition International LLC, Brentwood, MO, USA) and water. The rats were housed with a 12 h light and 12 h dark cycle. All of the experimental protocols involving animals were reviewed and approved by the Institutional Animal Care and Use Committee (IACUC number: 981106) of National Yang-Ming University. Rats were anesthetized with urethane (1 g/kg, i.p.). All of the animals were equipped with the left femoral vein for solution or drug administration.

The rats were intravenously infused with TPN via the left femoral artery at a flow rate of 20 mL/kg/h for 3 h to induce acute liver injury. Normal saline (0.9% sodium chloride) was used as the vehicle for the control group. Different doses of glycyrrhizin (1, 3 or 10 mg/kg) were administered intravenously before TPN infusion.

Before TPN administration, glycyrrhizin was dissolved in 1 mL normal saline and given as an i.v. bolus, whereas the TPN group received an equal volume of normal saline (1 mL via i.v. bolus). The total energy of Kabiven^®^ was 1038 kcal [11% glucose, 885 mL; amino acids/electrolytes (Vamin 18), 300 mL; and 20% fat emulsion (Intralipid), 255 mL]. Each rat received a total infusion volume of 12 mL TPN at a rate of 20 mL/kg/h for three h. A typical 200 mg rat received approximately 8.6 kcal of energy.

Every group had six animals. The blood samples were collected through left femoral vein immediately and stored in heparin-coated capillary tubes until liver enzyme profiling and biochemical analyses. The liver tissue was collected, washed with normal saline and then fixed in 10% neutral buffered formalin for histopathological examinations.

### 4.3. Biochemical Analysis

The blood samples were collected from the animals and centrifuged at 3000 rpm for 15 min. The plasma samples were used for the following biochemical analyses of aspartate aminotransferase (AST), alanine aminotransferase (ALT), total bilirubin (TB) and triglyceride (TG) levels. All of these analyses were performed using the Hitachi 902 Automatic Analyzer with adapted reagents from Roche (Boehringer, Mannheim, Germany).

### 4.4. Histopathological Evaluation

The liver tissues were fixed in 10% buffered formalin, embedded in paraffin and cut into 4 μm-thick slides transversely from portal Zone 1 to Zone 3, which is close to the terminal of hepatic venule tract, for hematoxylin and eosin (H & E) staining (Tokyo, Japan). The rest of tissues were stored at −80 °C. The histopathological changes in liver cell morphology were examined using a BX51 light microscope (Olympus, Tokyo, Japan) at high power (200× magnification) for each slide. For quantitative purposes, two portal zones were randomly chosen in each slide and photographed using an Image-pro Plus medical image analysis system. The cell numbers were recorded. The normal hepatocytes were counted from 10 fields that were randomly chosen in liver samples from each group under 200× magnification. The means numbers of normal cells were calculated per microscope field from six animals in each group.

### 4.5. Immunohistochemical Assay

Immunohistochemistry (IHC) staining was performed using a biotinylated secondary antibody (Vectastain Universal Elite ABC Kit, Burlingame, CA, USA). Monoclonal rabbit antibodies against human SOCS3 and cleaved-caspase 3 were diluted at a ratio of 1:100. The primary antibodies were omitted for the negative controls. For three slides, the cytoplasm stained brown and was scored positive. The expression of SOCS3 and cleaved-caspase 3 was quantitatively evaluated using an Olympus C × 31 microscope with the Image-pro Plus medical image analysis system. The digital images were captured using a digital camera (Canon A640, Tokyo, Japan). The positive area and optical density (OD) of SOCS3 and cleaved-caspase 3-positive cells were determined by measuring three randomly selected microscopic fields (400× magnification) for each slide. The IHC index was defined as average integral optical density (AIOD) (AIOD = positive area × OD/total area).

### 4.6. Western Blot Analysis

After sacrifice, the rats’ livers were rapidly removed, disassociated and homogenized in ice-cold lysis buffer (1% NP-40, 0.5% sodium deoxycholate, 0.1% sodium dodecyl sulfate and a protease inhibitor mixture comprised of phenylmethylsulfonyl fluoride, aprotinin and sodium orthovanadate). The total cell lysate (50 μg of proteins) was separated by the SDS-polyacrylamide gel electrophoresis (PAGE) (12% running and 4% stacking) and analyzed using the designated antibodies. The Western-Light Chemiluminescent detection system (Bio-Rad, Hercules, CA, USA) was used to detect the signals, as previously described by Chiu *et al.* [54].

### 4.7. Statistical Analyses

The data are expressed as the mean ± standard deviation (SD) of three independent experiments, and all of the data were analyzed using one-way analysis of variance (ANOVA) with Bonferroni correction. The data were analyzed using the SAS software statistical package “SigmaPlot” version 9.0 (SAS Institute Inc., Cary, NC, USA).

## 5. Conclusions

Taken together, our data provide new information on the molecular mechanisms by which glycyrrhizin inhibits inflammation. Namely, GL acts by reducing iNOS, TNF-α, IL-1β, IL-6 and nitrotyrosine levels through the activation of SOCS3. In addition, glycyrrhizin was found to have anti-apoptotic effects on TPN-related acute liver injury in rats through the inhibition of JNK1/2 and p38 MAPK phosphorylation and inactivation of the CHOP protein, thereby reducing ER stress. The inhibitory effect of glycyrrhizin on ER stress and reactive nitrogen species production is one possible mechanism by which glycyrrhizin prevents TPN-related liver injury. Overall, we demonstrated a novel animal model for early stage of TPN related acute liver injury and elucidated the potential therapeutic effects of glycyrrhizin on TPN related hepatitis. Nevertheless, glycyrrhizin therapy for chronic parenteral nutrition-related hepatitis warrants further investigation.

## Figures and Tables

**Table 1:** Effect of GL on the serum levels of liver enzymes, triglyceride and total bilirubin in total parenteral nutrition (TPN) rats.

Group	ALT (U/L)	AST (U/L)	TB (mg/dL)	TG (μg/μL)
Control	27 ±4	123 ±6	0.10 ±0.1	5.5 ±0.4
TPN	55 ±5 *	434 ±5 *	0.41 ±0.1 *	9.3 ±0.4 *
GL 1 mg/kg + TPN	38 ±4 #	310 ±16 #	0.36 ±0.1 *	8.1 ±0.4 #
GL 3 mg/kg + TPN	33 ±3 #	236 ±9 #	0.26 ±0.1	6.9 ±0.3 #
GL 10 mg/kg + TPN	24 ±3 #	146 ±9 #	0.20 ±0.4	6.4 ±0.3 #

Values represent the mean ± SD (*n =* 6);

* *p <* 0.05 (*versus* control group);

# *p <* 0.05 (between TPN and GL-pretreated + TPN groups).

ALT: alanine aminotransferase; AST: aspartate aminotransferase; TB: total bilirubin; TG: triglyceride.

## References

[1] Chance W.T., Sheriff S., Dayal R., Friend L.A., Thomas I., Balasubramaniam A. (2006). The role of polyamines in glucagon-like peptide-2 prevention of TPN-induced gut hypoplasia. Peptides.

[2] Stoll B., Horst D.A., Cui L., Chang X., Ellis K.J., Hadsell D.L., Suryawan A., Kurundkar A., Maheshwari A., Davis T.A. (2010). Chronic parenteral nutrition induces hepatic inflammation, steatosis, and insulin resistance in neonatal pigs. J. Nutr.

[3] Raman M., Allard J.P. (2007). Parenteral nutrition related hepato-biliary disease in adults. Appl. Physiol. Nutr. Metable.

[4] Sandhu I.S., Jarvis C., Everson G.T. (1999). Total parenteral nutrition and cholestasis. Clin. Liver Dis.

[5] Kumpf V.J. (2006). Parenteral nutrition-associated liver disease in adult and pediatric patients. Nutr. Clin. Pract.

[6] Cavicchi M., Beau P., Crenn P., Degott C., Messing B. (2000). Prevalence of liver disease and contributing factors in patients receiving home parenteral nutrition for permanent intestinal failure. Ann. Intern. Med.

[7] Luman W., Shaffer J.L. (2002). Prevalence, outcome and associated factors of deranged liver function tests in patients on home parenteral nutrition. Clin. Nutr.

[8] Attili A.F., Angelico M., Cantafora A., Alvaro D., Capocaccia L. (1986). Bile acid-induced liver toxicity: Relation to the hydrophobic-hydrophilic balance of bile acids. Med. Hypotheses.

[9] Tomar B.S. (2000). Hepatobiliary abnormalities and parenteral nutrition. Indian J. Pediatr.

[10] Brinkman A.S., Murali S.G., Hitt S., Solverson P.M., Holst J.J., Ney D.M. (2012). Enteral nutrients potentiate glucagon-like peptide-2 action and reduce dependence on parenteral nutrition in a rat model of human intestinal failure. Am. J. Physiol. Gastrointest Liver Physiol.

[11] Jain A.K., Stoll B., Burrin D.G., Holst J.J., Moore D.D. (2012). Enteral bile acid treatment improves parenteral nutrition-related liver disease and intestinal mucosal atrophy in neonatal pigs. Am. J. Physiol. Gastrointest Liver Physiol.

[12] Loff S., Kranzlin B., Moghadam M., Dzakovic A., Wessel L., Back W., Hosie S., Wirth H., Waag K.L. (1999). Parenteral nutrition-induced hepatobiliary dysfunction in infants and prepubertal rabbits. Pediatr. Surg. Int.

[13] Shamir R., Zahavi I., Bar-Sever Z., Heckelman B., Marcus H., Dinari G. (1993). Total parenteral nutrition-associated cholestasis after selective damage to acinar zone 3 hepatocytes by bromobenzene in the rat. Life Sci.

[14] Zahavi I., Rosezki O., Stolkart Y., Shamir R., Heckelman B., Marcus H., Dinari G. (2000). The effect of cisapride on total parenteral nutrition-associated cholestasis in rats. Isr. Med. Assoc. J.

[15] Hong L., Wang X., Wu J., Cai W. (2009). Mitochondria-initiated apoptosis triggered by oxidative injury play a role in total parenteral nutrition-associated liver dysfunction in infant rabbit model. J. Pediatr. Sur.

[16] Laborie S., Lavoie J.C., Chessex P. (1998). Paradoxical role of ascorbic acid and riboflavin in solutions of total parenteral nutrition: Implication in photoinduced peroxide generation. Pediatr. Res.

[17] Knowles H., Li Y., Perraud A.L. (2013). The TRPM2 ion channel, an oxidative stress and metabolic sensor regulating innate immunity and inflammation. Immunol. Res.

[18] Egwuagu C.E., Yu C.R., Zhang M., Mahdi R.M., Kim S.J., Gery I. (2002). Suppressors of cytokine signaling proteins are differentially expressed in Th1 and Th2 cells: Implications for Th cell lineage commitment and maintenance. J. Immunol.

[19] Yu C.R., Mahdi R.M., Ebong S., Vistica B.P., Chen J., Guo Y., Gery I., Egwuagu C.E. (2004). Cell proliferation and STAT6 pathways are negatively regulated in T cells by STAT1 and suppressors of cytokine signaling. J. Immunol.

[20] Yoshimura A., Suzuki M., Sakaguchi R., Hanada T., Yasukawa H. (2012). SOCS, Inflammation, and autoimmunity. Front. Immunol.

[21] Tazuke Y., Drongowski R.A., Btaiche I., Coran A.G., Teitelbaum D.H. (2004). Effects of lipid administration on liver apoptotic signals in a mouse model of total parenteral nutrition (TPN). Pediatr. Surg. Int.

[22] Ferri K.F., Kroemer G. (2001). Organelle-specific initiation of cell death pathways. Nat. Cell. Biol.

[23] Wang H.C., Huang W., Lai M.D., Su I.J. (2006). Hepatitis B virus pre-S mutants, endoplasmic reticulum stress and hepatocarcinogenesis. Cancer Sci.

[24] Katz M.S., Thatch K.A., Schwartz M.Z. (2010). Dose variation of hepatocyte growth factor and its effects on an animal model of TPN-induced liver injury. J. Surg. Res.

[25] Liu J., Ren F., Cheng Q., Bai L., Shen X., Gao F., Busuttil R.W., Kupiec-Weglinski J.W., Zhai Y. (2012). Endoplasmic reticulum stress modulates liver inflammatory immune response in the pathogenesis of liver ischemia and reperfusion injury. Transplantation.

[26] Ashfaq U.A., Masoud M.S., Nawaz Z., Riazuddin S. (2011). Glycyrrhizin as antiviral agent against Hepatitis C Virus. J. Transl. Med.

[27] Liao H.L., Ma T.C., Li Y.C., Chen J.T., Chang Y.S. (2010). Concurrent use of corticosteroids with licorice-containing TCM preparations in Taiwan: A National Health Insurance Database study. J. Altern. Complement. Med.

[28] Asl M.N., Hosseinzadeh H. (2008). Review of pharmacological effects of *Glycyrrhiza* sp. and its bioactive compounds. Phytother. Res.

[29] Del Prete A., Scalera A., Iadevaia M.D., Miranda A., Zulli C., Gaeta L., Tuccillo C., Federico A., Loguercio C. (2012). Herbal products: Benefits, limits, and applications in chronic liver disease. Evid. Based Complement. Alternat. Med.

[30] Stickel F., Schuppan D. (2007). Herbal medicine in the treatment of liver diseases. Dig. Liver Dis.

[31] Makuuchi M., Kokudo N., Arii S., Futagawa S., Kaneko S., Kawasaki S., Matsuyama Y., Okazaki M., Okita K., Omata M. (2008). Development of evidence-based clinical guidelines for the diagnosis and treatment of hepatocellular carcinoma in Japan. Hepatol. Res.

[32] Van der kallen C.J., van greevenbroek M.M., Stehouwer C.D., Schalkwijk C.G. (2009). Endoplasmic reticulum stress-induced apoptosis in the development of diabetes: Is there a role for adipose tissue and liver?. Apoptosis.

[33] Farley N., Pedraza-Alva G., Serrano-Gomez D., Nagaleekar V., Aronshtam A., Krahl T., Thornton T., Rincón M. (2006). p38 mitogen-activated protein kinase mediates the Fas-induced mitochondrial death pathway in CD8+ T cells. Mol. Cell. Biol.

[34] McGuinness O.P., Donmoyer C., Ejiofor J., McElligott S., Lacy D.B. (1998). Hepatic and muscle glucose metabolism during total parenteral nutrition: Impact of infection. Am. J. Physiol.

[35] Jiménez W., Clária J., Arroyo V., Rodés J. (1992). Carbon tetrachloride induced cirrhosis in rats: A useful tool for investigating the pathogenesis of ascites in chronic liver disease. J. Gastroenterol. Hepatol.

[36] Manns M.P., Wedemeyer H., Singer A., Khomutjanskaja N., Dienes H.P., Roskams T., Goldin R., Hehnke U., Inoue H., European SNMC Study Group (2012). Glycyrrhizin in patients who failed previous interferon alpha-based therapies: Biochemical and histological effects after 52 weeks. J. Viral Hepat.

[37] Yoshikawa M., Matsui Y., Kawamoto H., Umemoto N., Oku K., Koizumi M., Yamao J., Kuriyama S., Nakano H., Hozumi N. (1997). Effects of glycyrrhizin on immune-mediated cytotoxicity. J. Gastroenterol. Hepatol.

[38] Kao T.C., Shyu M.H., Yen G.C. (2010). Glycyrrhizic acid and 18beta-glycyrrhetinic acid inhibit inflammation via PI3K/Akt/GSK3beta signaling and glucocorticoid receptor activation. J. Agric. Food Chem.

[39] Wei X.Q., Charles I.G., Smith A., Ure J., Feng G.J., Huang F.P., Xu D., Muller W., Moncada S., Liew F.Y. (1995). Altered immune responses in mice lacking inducible nitric oxide synthase. Nature.

[40] Nussler A.K., Billiar T.R. (1993). Inflammation, immunoregulation, and inducible nitric oxide synthase. J. Leukoc. Biol.

[41] Minc-Golomb D., Tsarfaty I., Schwartz J.P. (1994). Expression of inducible nitric oxide synthase by neurones following exposure to endotoxin and cytokine. Br. J. Pharmacol.

[42] Ialenti A., Ianaro A., Moncada S., di Rosa M. (1992). Modulation of acute inflammation by endogenous nitric oxide. Eur J. Pharmacol.

[43] Fan C.K., Lin Y.H., Hung C.C., Chang S.F., Su K.E. (2004). Enhanced inducible nitric oxide synthase expression and nitrotyrosine accumulation in experimental granulomatous hepatitis caused by Toxocaracanis in mice. Parasite Immunol.

[44] Gorbunov N.V., McFaul S.J., Januszkiewicz A., Atkins J.L. (2005). Pro-inflammatory alterations and status of blood plasma iron in a model of blast-induced lung trauma. Int. J. Immunopathol. Pharmacol.

[45] Di Giannantonio M., Frydas S., Kempuraj D., Karagouni E., Hatzistilianou M., Conti C.M., Boucher W., Papadopoulou N., Donelan J., Cao J. (2005). Cytokines in stress. Int. J. Immunopathol. Pharmacol.

[46] Feng Y., Ralls M.W., Xiao W., Miyasaka E., Herman R.S., Teitelbaum D.H. (2012). Loss of enteral nutrition in a mouse model results in intestinal epithelial barrier dysfunction. Ann. N. Y. Acad. Sci.

[47] Feng Y., McDunn J.E., Teitelbaum D.H. (2010). Decreased phospho-Akt signaling in a mouse model of total parenteral nutrition: A potential mechanism for the development of intestinal mucosal atrophy. Am. J. Physiol. Gastrointest Liver Physiol.

[48] Koeberlein B., zur Hausen A., Bektas N., Zentgraf H., Chin R., Nguyen L.T., Kandolf R., Torresi J., Bock C.T. (2010). Hepatitis B virus overexpresses suppressor of cytokine signaling-3 (SOCS3) thereby contributing to severity of inflammation in the liver. Virus Res.

[49] Sasaki A., Yasukawa H., Shouda T., Kitamura T., Dikic I., Yoshimura A. (2000). CIS3/SOCS-3 suppresses erythropoietin (EPO) signaling by binding the EPO receptor and JAK2. J. Biol. Chem.

[50] Boden G., Song W., Duan X., Cheung P., Kresge K., Barrero C., Merali S. (2011). Infusion of glucose and lipids at physiological rates causes acute endoplasmic reticulum stress in rat liver. Obesity.

[51] Kamiya T., Nishihara H., Hara H., Adachi T. (2012). Ethanol extract of Brazilian red propolis induces apoptosis in human breast cancer MCF-7 cells through endoplasmic reticulum stress. J. Agric. Food Chem.

[52] Malhi H., Kaufman R.J. (2011). Endoplasmic reticulum stress in liver disease. J. Hepatol.

[53] Riedl S.J., Shi Y. (2004). Molecular mechanisms of caspase regulation during apoptosis. Nat. Rev. Mol. Cell. Biol.

[54] Yeh Y.T., Hur S.S., Chang J., Wang K.C., Chiu J.J., Li Y.S., Chien S. (2012). Matrix stiffness regulates endothelial cell proliferation through Septin 9. PLoS One.

